# Supplementary material for: Conditional chemoconnectomics (cCCTomics) as a strategy for efficient and conditional targeting of chemical transmission
Source: eLife. 2024 Apr 30;12:RP91927. doi: 10.7554/eLife.91927 (PMC11060718; doi:10.7554/eLife.91927)
Supplement: Figure 3—source data 3. [file elife-91927-fig3-data3.zip › Figure 3 source data3 part 2.zip!/Figure 3-Source data 3-part2/1063-1-CRISPResso_Report_az97gqtx.zip!/Nucleotide_percentage_quilt.pdf]

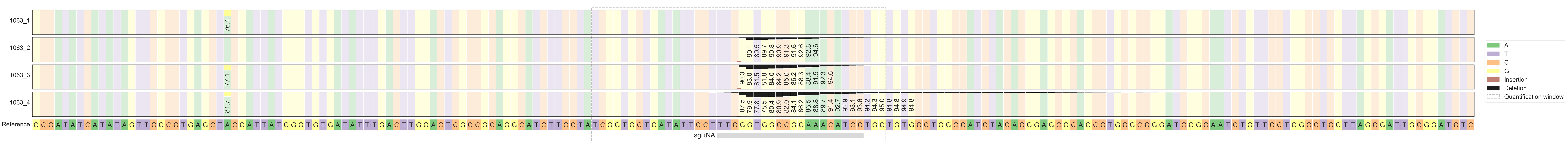
1063_1
76.4
1063_2
90.1 89.5 89.7 90.8 90.9 91.3 91.6 92.6 92.8 94.6
1063_3
77.1
90.3 83.0 81.5 81.8 84.0 84.2 85.0 86.2 88.3 88.4 91.5 92.3 94.6
1063_4
81.7
87.5 79.9 77.8 78.5 80.4 80.9 82.0 84.1 86.2 86.5 88.8 89.7 91.4 92.7 92.9 93.1 93.6 94.2 94.3 95.0 94.8 94.8 94.9 94.8
Reference
GCCATATCATATAGTTCGCCTGAGCTACGATTATGGGTGTGATATTTGACTTGGACTCGCCGCAGGCATCTTCCTATCGGTGCTGATATTCCTTTCGGTGGCCGGAAACATCCTGGTGTGCCTGGCCATCTACACGGAGCGCAGCCTGCGCCGGATCGGCAATCTGTTCCTGGCCTCGTTAGCGATTGCGGATCTC
sgRNA
A
T
C
G
Insertion
Deletion
Quantification window
